# Association Between Post-COVID-19 Herpes Zoster Reactivation and Peripheral Nervous System Disorders: Multinational Real-World Evidence from TriNetX

**DOI:** 10.7150/ijms.127617

**Published:** 2026-04-23

**Authors:** Yu-Chen Cheng, Chien-Lin Lu, Joshua Wang, Yi-Chou Hou, Kuo-Cheng Lu

**Affiliations:** 1 School of Medicine, College of Medicine, Fu Jen Catholic University, New Taipei City, Taiwan; 2 Department of Neurology, Fu Jen Catholic University Hospital, Fu Jen Catholic University, New Taipei City, Taiwan; 3 Division of Nephrology, Department of Internal Medicine, Fu Jen Catholic University Hospital, Fu Jen Catholic University, New Taipei City 24352, Taiwan.; 4 Department of Research, Taipei Tzu Chi Hospital, Buddhist Tzu Chi Medical Foundation, New Taipei City, Taiwan.; 5 School of Biomedical Sciences, Queensland University of Technology, Brisbane, Australia.; 6 Division of Nephrology, Department of Internal Medicine, Cardinal-Tien Hospital, School of Medicine, College of Medicine, Fu Jen Catholic University, New Taipei City 24205, Taiwan.; 7 Division of Nephrology, Department of Medicine, Taipei Tzu Chi Hospital, Buddhist Tzu Chi Medical Foundation, New Taipei City, Taiwan.

**Keywords:** autoimmune diseases, Bell's palsy, COVID-19, Guillain-Barré syndrome, herpes zoster, myasthenia gravis, peripheral nervous system, SARS-CoV-2

## Abstract

**Background:**

The increased incidence of herpes zoster (HZ) following coronavirus disease 2019 (COVID-19) suggests a period of immune dysregulation, but the associated long-term neuro-immunological risks remain unclear.

**Methods:**

Utilizing de-identified electronic health records from the TriNetX Global Collaborative Network, we established 1:1 propensity score-matched cohorts of COVID-19 survivors, comparing HZ-exposed and unexposed groups over a three-year follow-up. Primary endpoints included the incidence of peripheral nervous system disorders: Bell's palsy, Guillain-Barré syndrome (GBS), and myasthenia gravis (MG). Analyses included Cox proportional hazards models, landmark analyses, and multiple sensitivity analyses.

**Results:**

HZ reactivation was associated with increased three-year risks of all outcomes. The risk of Bell's palsy was elevated early and remained sustained (hazard ratio 3.625, 95% confidence interval 3.151-4.170). In contrast, the risks of GBS (hazard ratio 1.858, 95% confidence interval 1.243-2.779) and MG (hazard ratio 1.640, 95% confidence interval 1.178-2.284) showed delayed increases emerging after the first year. These associations remained consistent across sensitivity analyses and were more pronounced in individuals with metabolic comorbidities. COVID-19 vaccination was not associated with an increased risk of these outcomes, although subgroup findings should be interpreted with caution due to limited event counts.

**Conclusions:**

Post-COVID-19 HZ is associated with an increased risk of peripheral nervous system disorders, highlighting the need for symptom-based neurological awareness during both early and delayed post-infectious periods.

## Introduction

Coronavirus disease 2019 (COVID-19) profoundly impacts the peripheral nervous system (PNS) 1. A prominent complication is increased herpes zoster (HZ) reactivation 2. HZ represents clinical reactivation of latent varicella-zoster virus (VZV) within sensory ganglia, producing characteristic vesicular rash and neuronal damage 3.

Observational studies confirm substantially elevated HZ risk in COVID-19 survivors versus uninfected controls (reported hazard ratios [HRs]: 1.59-2.16) 4, 5. SARS-CoV-2 likely induces immunological perturbations—including lymphopenia and impaired VZV-specific cell-mediated immunity 2, 6, 7 —while Th17/IL-17 pathway activation may further predispose to VZV reactivation 8.

HZ's neurotropism frequently causes direct PNS pathology, including cranial nerve involvement 2, 3. Ramsay Hunt syndrome (Bell's palsy variant) exemplifies VZV-mediated facial paralysis 9, 10. Post-COVID HZ may thus compound SARS-CoV-2's direct neuroinflammatory effects 11, 12.

COVID-19 independently elevates risks of Guillain-Barré syndrome (GBS; post-infectious demyelination) 13, 14, myasthenia gravis (MG; molecular mimicry) 15, 16 and Bell's palsy 17. Although literature links COVID-19 to HZ, GBS, and MG 16-18, HZ's mediating role remains unexplored 16, 18, 19.

Given HZ's immune dysregulation and neural inflammation, we hypothesize that post-COVID HZ reactivation exacerbates latent immune dysfunction, increasing subsequent autoimmune/inflammatory PNS disorder risk. This propensity score-matched (PSM) cohort study therefore evaluates Bell's palsy, GBS, and MG incidence in COVID-19 survivors with versus without subsequent HZ over a three-year follow-up.

## Materials and Methods

### Study design and data source

We conducted a retrospective cohort study using the TriNetX federated global research network (Global Collaborative Network), which provides access to de-identified electronic health records (EHRs) from 144 international healthcare organizations. The database encompasses structured patient dossiers including demographics, clinical diagnoses (International Classification of Diseases, Tenth Revision, Clinical Modification [ICD-10-CM]), procedures, medications, and laboratory results. This study followed the Strengthening the Reporting of Observational Studies in Epidemiology (STROBE) reporting guidelines and the tenets of the Declaration of Helsinki. Institutional Review Board (IRB) approval was obtained from Taipei Tzu Chi Hospital (No: 14-IRB140), with a waiver of informed consent due to the use of de-identified retrospective data.

### Study population

Adult patients (aged ≥ 18 years) with a confirmed COVID-19 diagnosis between January 1, 2020, and December 31, 2022, were identified. COVID-19 was defined by a positive SARS-CoV-2 RNA laboratory result (TriNetX code: 9088) or ICD-10-CM code U07.1. The index date was defined as the date of the first recorded COVID-19 diagnosis or positive laboratory test. From the initial pool of COVID-19 survivors, we identified an exposed group (COVID-19 + HZ; n = 110,054) comprising patients with a new diagnosis of HZ (ICD-10-CM B02 and subtypes) occurring between day 1 and 3 years following the index date, and an unexposed group (COVID-19 only; n = 14,849,703) comprising patients without HZ during the follow-up. Patients with a history of HZ within one year prior to or on the index date were excluded.

### Outcome measures

The primary outcomes were the 3-year incidence of three PNS disorders: Bell's palsy (ICD-10 G51.0), GBS (ICD-10 G61.0), and MG (ICD-10 G70.0, G70.00, G70.01). To ensure all outcomes were incident events, patients with a documented history of the specific outcome prior to the index date were excluded from the respective analysis. Follow-up commenced one day after the index date and continued for up to 1,095 days, terminating at the occurrence of the outcome, death, or loss to follow-up.

### Propensity score matching

To address potential confounding and the computational constraints of the large-scale dataset, a two-stage 1:1 PSM strategy was employed using a greedy nearest-neighbor algorithm. The primary analysis matched the overall cohort on essential covariates, including age, sex, White race, diabetes mellitus, and hypertension. A complementary validation was conducted on the age-restricted cohort (50-59 years) to allow for a broader adjustment of clinical and laboratory parameters. Covariate balance was defined by a standardized mean difference (SMD) < 0.1.

### Statistical analysis

Following PSM, the analysis involved two distinct approaches. First, absolute risks, relative risks (RRs), and odds ratios (ORs) were calculated for the PNS outcomes (Supplementary Table S2). Second, a time-to-event analysis was performed using Kaplan-Meier survival curves (log-rank test) and Cox proportional hazards models to estimate HRs and 95% confidence intervals (CIs). The proportionality assumption of the Cox model was formally evaluated. Statistical significance was predetermined at a two-sided p-value < 0.05.

### Sensitivity analysis

To ensure the reliability of the findings, several sensitivity analyses were conducted. These included comparisons between the age-restricted cohort (50-59 years) and the overall cohort to assess generalizability. Temporal stability and sequencing were evaluated using landmark analyses and time-window analyses, with additional exclusion of individuals whose outcomes occurred prior to or concurrent with HZ onset. Clinical sub-analyses were performed by excluding individuals with immunosuppression, human immunodeficiency virus (HIV) infection (ICD-10: B20), or influenza (ICD-10: J09-J11), and by evaluating the influence of vaccination. To address potential detection bias, diagnostic frequency, nerve conduction velocity (NCV) utilization, and hospitalization burden were assessed. Finally, additional analyses examined the impact of anti-HZ therapy and calculated E-values to evaluate the influence of unmeasured confounding.

## Results

### Cohort characteristics and baseline balance

The baseline characteristics of the age-restricted cohort (50-59 years) before and after PSM are presented in Table 1. Prior to matching, the COVID+HZ group exhibited higher proportions of females (64.4% vs. 53.1%), White race (68.5% vs. 57.8%), hypertension (27.2% vs. 16.0%), and diabetes mellitus (14.3% vs. 8.0%). After 1:1 PSM, all covariates achieved balance with standardized mean differences (SMD) < 0.10. Similar baseline patterns and post-match balance were observed in the overall cohort (18-89 years) (Supplementary Table S1).

### Bell's palsy

**Figure 2:**
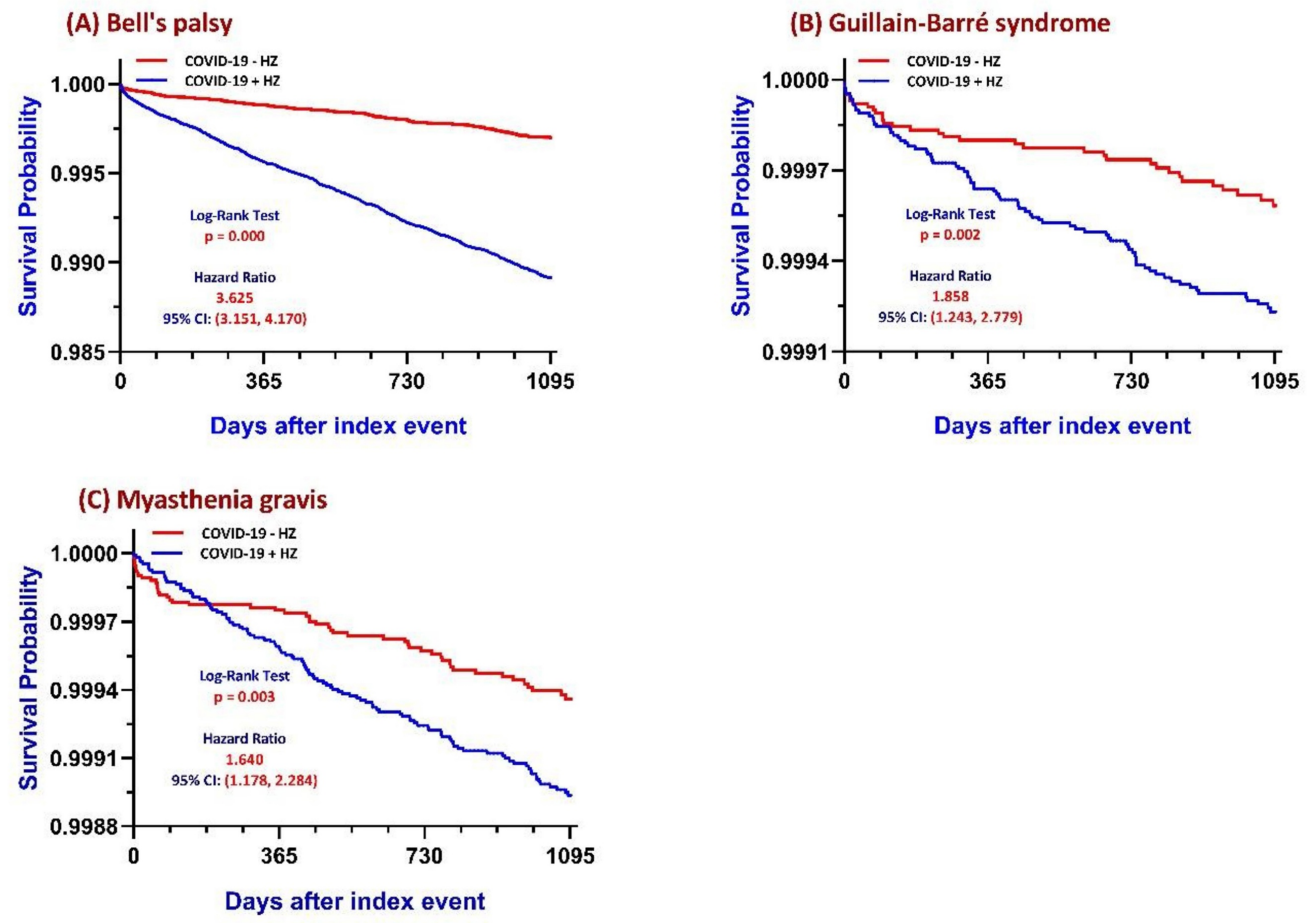
** Kaplan-Meier survival curves comparing COVID-19 patients with and without Herpes Zoster (HZ) reactivation.** Cumulative incidence of freedom from (A) Bell's palsy, (B) Guillain-Barré syndrome, and (C) myasthenia gravis over a 3-year follow-up period is shown for the HZ-exposed and matched control cohorts. P values were calculated using the log-rank test. **Abbreviations:** HZ, herpes zoster; COVID-19, coronavirus disease 2019; COVID+ HZ, COVID-19 survivors with HZ reactivation; COVID- HZ, COVID-19 survivors without HZ reactivation.

**Figure 3:**
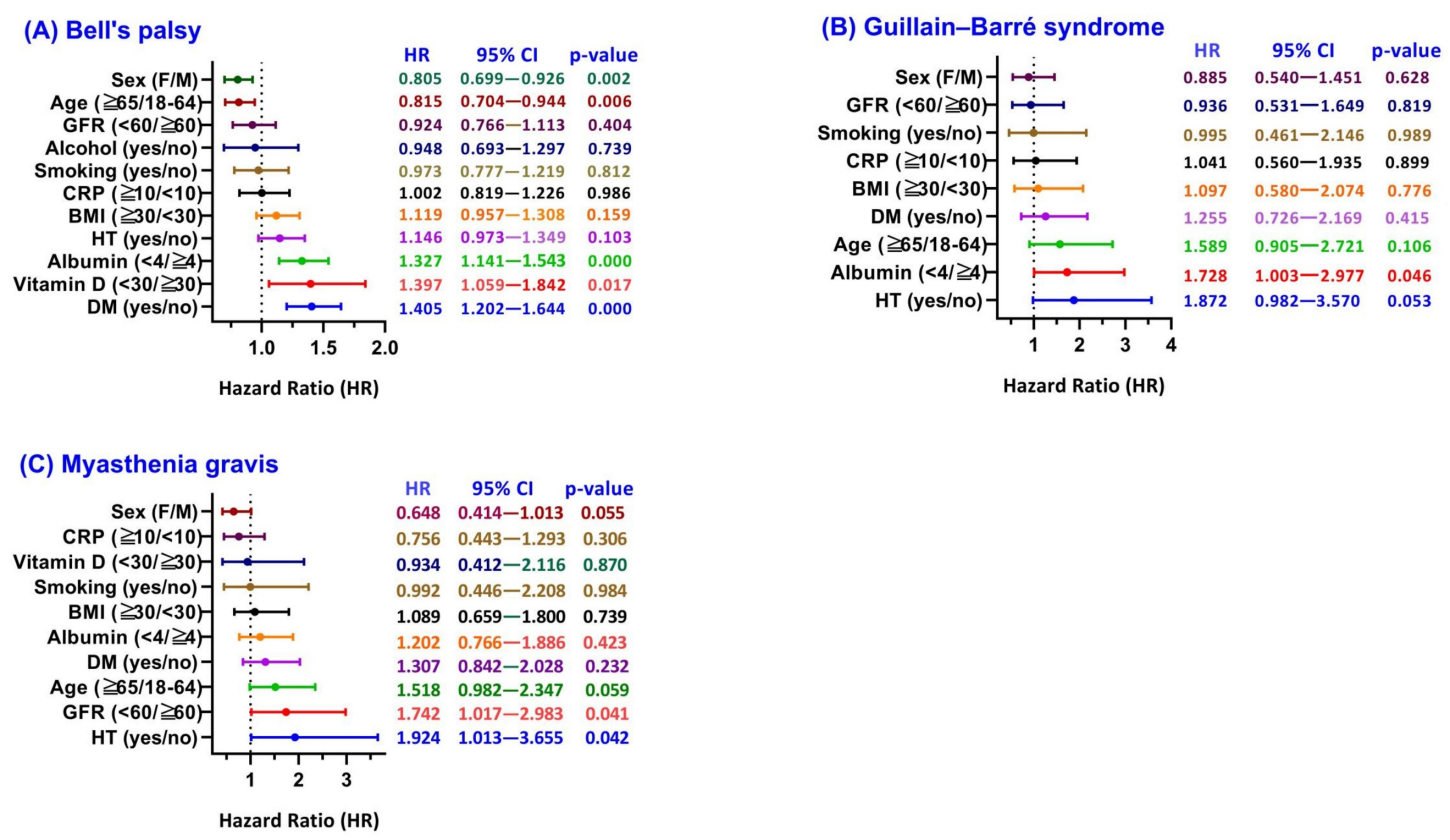
** Subgroup analysis of Peripheral Nervous System outcomes: hazard ratios associated with Herpes Zoster (HZ).** Forest plots showing hazard ratios (HRs) for (A) Bell's palsy, (B) Guillain-Barré syndrome, and (C) Myasthenia gravis, comparing HZ-exposed versus HZ-unexposed COVID-19 survivors stratified by baseline factors. Data are presented as adjusted HRs with 95% confidence intervals (CIs). **Abbreviations:** HZ, herpes zoster; COVID-19, coronavirus disease 2019; GFR, glomerular filtration rate; CRP, C-reactive protein; BMI, body mass index; HT, hypertension; DM, diabetes mellitus; HR, hazard ratio; CI, confidence interval.

Among COVID-19 survivors, the COVID+HZ group (n = 109,010) demonstrated a higher 3-year cumulative incidence of Bell's palsy compared to the COVID-HZ group (1.00% vs. 0.22%; absolute risk difference 0.008, 95% CI 0.007-0.009; p < 0.001). Time-to-event analysis yielded a Cox HR of 3.625 (95% CI 3.151-4.170; log-rank p < 0.001) (Figure 2A; Supplementary Table S2). Subgroup analysis (Figure 3A) revealed that metabolic vulnerabilities were associated with further elevated HRs, including diabetes (HR 1.405, 95% CI 1.202-1.644; p < 0.001), vitamin D deficiency (HR 1.397, 95% CI 1.059-1.842; p = 0.017), and hypoalbuminemia (HR 1.327, 95% CI 1.141-1.543; p < 0.001). Conversely, lower HRs were observed in older adults (≥ 65 years: HR 0.815, 95% CI 0.704-0.944; p = 0.006) and females (HR 0.805, 95% CI 0.699-0.926; p = 0.002).

### Guillain-Barré syndrome and myasthenia gravis

The 3-year GBS incidence was 0.07% (79/109,918) in the COVID+HZ group versus 0.03% (34/109,975) in the COVID-HZ group (RR 2.33, 95% CI 1.56-3.48; Supplementary Table S2). Kaplan-Meier curves showed early separation (log-rank χ² = 9.406, p = 0.002; Figure 2B), with a Cox HR of 1.858 (95% CI 1.243-2.779). For MG, subsequent HZ was associated with a higher 3-year incidence (0.10% vs. 0.05%; risk difference 0.001, 95% CI 0.000-0.001; p < 0.001), with a Cox HR of 1.640 (95% CI 1.178-2.284; Figure 2C). Subgroup analyses (Figure 3B, 3C) identified higher risks associated with hypertension (GBS HR 1.872; MG HR 1.924) and reduced kidney function (MG HR 1.742, 95% CI 1.017-2.983).

### Comprehensive sensitivity and robustness analyses

Landmark analysis (Supplementary Table S3) showed distinct temporal patterns, with an immediate elevation in Bell's palsy risk (HR approximately 3.6-3.8 across time points), whereas associations for GBS and MG were not significant at 1 year but became significant during longer follow-up periods. Time-window-based analysis showed the HR for Bell's palsy peaked within the first 6 months (HR 5.02, 95% CI 3.629-6.942) (Supplementary Table S9). To ensure temporal sequence, a sensitivity analysis excluding individuals whose PNS outcomes occurred prior to or concurrent with their first HZ episode was performed (Supplementary Table S10); the risk for Bell's palsy remained significant (HR 1.428, 95% CI 1.203-1.696), while associations for GBS and MG were attenuated. E-value analysis yielded values of 6.72 for Bell's palsy, 3.12 for GBS, and 2.66 for MG (Supplementary Table S14).

The exclusion of pre-index immunosuppression or radiotherapy resulted in HRs of 3.74 for Bell's palsy and 2.51 for GBS (Supplementary Table S4). After excluding individuals with HIV or influenza, HRs remained significant: 4.17 (95% CI 3.52-4.95) for Bell's palsy, 2.32 (95% CI 1.38-3.91) for GBS, and 1.53 (95% CI 1.04-2.26) for MG (Supplementary Table S6). Vaccination-stratified analyses (Supplementary Tables S5A and S5B) showed that Bell's palsy risk persisted in both vaccinated (HR 3.685, 95% CI 2.674-5.077) and unvaccinated (HR 4.138, 95% CI 3.514-4.873) cohorts. Additional sensitivity analysis for HZ vaccination status demonstrated consistent risk for Bell's palsy (Supplementary Table S13).

### Detection bias and treatment impact

Diagnostic frequency analysis: the average number of Bell's palsy-related diagnoses per patient was 1.291 ± 1.34 in the COVID+HZ group versus 1.521 ± 1.62 in the COVID-HZ group, with diagnostic ratios consistently exceeding 1.0 across all studied outcomes (Supplementary Table S7). Regarding the neurologic testing workout, the COVID+HZ cohort demonstrated significantly higher NCV utilization (mean 0.054 ± 0.45 vs. 0.022 ± 0.28; p < 0.001; Supplementary Table S8). Furthermore, the HZ-exposed group exhibited a higher 3-year hospitalization burden (mean 2.532 vs. 1.446, p < 0.0001; Supplementary Table S11). Finally, the impact of anti-HZ therapy on the 3-year risk of Bell's palsy among patients with concurrent infections showed no statistically significant reduction (HR 1.378, 95% CI 0.773-2.457; p = 0.275; Supplementary Table S12).

## Discussion

### Principal findings

This large-scale, longitudinal study provides evidence that HZ reactivation following COVID-19 is associated with a clinically meaningful increase in risk of subsequent neurological outcomes. Our 3-year analysis demonstrates a substantial relative risk increase across all primary outcomes: Bell's palsy showed the strongest association (HR 3.625), followed by GBS (HR 1.858) and MG (HR 1.640). While the absolute risks observed are relatively low (ranging from 0.07% to 1.00%), the observed associations may still be clinically relevant, particularly given the severity of these conditions, including the life-threatening nature of GBS 20 and the chronic management required for MG 21, as well as the large population of COVID-19 survivors.

Notably, the analysis revealed a distinct temporal divergence in risk profiles. Bell's palsy demonstrated an immediate and sustained risk elevation starting within the first six months, whereas the hazards for GBS and MG exhibited a delayed trajectory, becoming statistically prominent primarily after the first year. This temporal shift suggests a transition from acute viral-mediated injury 5 to a more chronic, secondary immune dysregulation 20, 22.

### Immediate vs. latent pathophysiological mechanisms

The distinct temporal patterns observed in our analysis provide important clues to the underlying mechanisms. The immediate increase in risk of Bell's palsy, occurring within the early post-HZ period, supports a mechanism related to direct viral neurotropism rather than a delayed immune-mediated process 11, 23. VZV reactivation within the geniculate ganglion may trigger acute neuritis and inflammatory edema 24, 25, which, within the anatomical confines of the fallopian canal 26, can result in facial nerve compression and ischemia 27, 28. Subgroup analyses further suggest that metabolic vulnerabilities, such as diabetes 10, 29 and vitamin D deficiency 30, may amplify this risk by impairing host immune responses and facilitating localized viral reactivation.

In contrast, the delayed emergence of GBS and MG, typically occurring beyond the first year, is more consistent with secondary immune-mediated mechanisms rather than direct viral invasion. Following HZ reactivation, a latency period may be required for the development of autoreactive immune responses 31, 32, such as molecular mimicry and epitope spreading 33-35. In GBS, this may involve immune-mediated injury to peripheral myelin 20, whereas in MG, disruption of neuromuscular transmission may occur through autoantibody-mediated mechanisms 22.

This temporal-mechanistic linkage is further supported by prior TriNetX analyses (e.g., Lu *et al*., Chien *et al*.), which demonstrated that HZ reactivation following COVID-19 serves as a systemic marker of post-viral immunodysregulation and is associated with an increased risk of subsequent multi-system complications, including cardiorenal and autoimmune sequelae 36, 37. Prior studies using artificial intelligence (AI)-based models have also highlighted the role of systemic inflammation in determining COVID-19 severity, which may contribute to subsequent immune dysregulation 38. The magnitude of systemic inflammation during acute COVID-19 may further influence this process by lowering the threshold for both VZV reactivation 6, 8 and subsequent neurological complications 39-41.

Together, these findings support a unified model in which HZ reactivation reflects a state of persistent immune dysregulation, predisposing individuals to both early neurotropic effects and delayed autoimmune-mediated complications.

### Clinical significance and stratified surveillance strategy

To translate these findings into clinical practice, we propose a stratified surveillance strategy tailored to high-risk populations, specifically those with advanced age or metabolic comorbidities. Given the temporal divergence observed, monitoring should be biphasic. During the acute phase (0-6 months), clinicians should prioritize cranial nerve assessments to ensure the timely administration of corticosteroids or antivirals for Bell's palsy, which may prevent irreversible axonal loss. For the latent phase (6-36 months), vigilance should shift toward detecting autoimmune markers or progressive neuromuscular symptoms. We specifically recommend strategic clinical checkpoints at 3, 12, and 24 months post-HZ. During these visits, patients should be screened for "red-flag" indicators such as progressive distal weakness, exercise-induced fatigue, or bulbar symptoms. Such a structured approach ensures that high-severity, low-frequency neuro-immunological sequelae are not overlooked during routine post-viral follow-up.

### Validation and internal consistency

The reliability of our findings is supported by several comprehensive sensitivity analyses. To establish stringent temporal precedence, we analyzed outcomes occurring prior to or on the same date as the HZ episode (Supplementary Table S10); the risk for Bell's palsy remained significant (HR 1.428; p < 0.0001), while GBS and MG risks decreased, suggesting those primary risks were driven by concurrent or temporally complex disease processes 28, 31, 41. To address potential detection bias, we verified that both groups averaged more than one ICD-10 entry per patient (1.291 vs. 1.521; Supplementary Table S7) and the COVID+HZ cohort demonstrated significantly higher NCV utilization (mean 0.054 vs. 0.022; p < 0.001; Supplementary Table S8), reflecting increased neurologic evaluation rather than administrative artifacts.

Notably, excluding individuals with prior immunosuppression or HIV infection had minimal impact on the results, reinforcing that the observed relationship persists in immunocompetent populations and is driven by VZV's inherent neurotropic capacity to reactivate during immune perturbation 42. Regarding vaccination, exploratory analyses showed that while COVID-19 vaccination did not fully mitigate the elevated neuro-immunological risk in HZ-exposed patients, the risks remained consistently high across both vaccinated and unvaccinated cohorts (HRs approximately 2.67-2.91), arguing against vaccine-induced injury as a primary driver 4. Furthermore, excluding individuals with a history of HZ vaccination did not significantly alter the HRs, indicating that the profound immune perturbation following COVID-19 may overcome the protection typically afforded by prior VZV-specific immunization. Additionally, the HZ-exposed group exhibited a markedly higher hospitalization burden (mean 2.532 vs. 1.446; p < 0.0001; Supplementary Table S11), underscoring that post-COVID HZ is associated with broader systemic morbidity 36, 43. Finally, anti-HZ therapy showed no statistically significant reduction in Bell's palsy risk (HR 1.378; p = 0.275; Supplementary Table S12), suggesting standard treatment alone may be insufficient to fully offset these risks.

### Limitations

Several limitations warrant consideration. First, as an observational cohort study, these findings demonstrate associations rather than definitive causation. While E-value analysis (Supplementary Table S14) suggests that an unmeasured confounder would need a very high effect size to nullify our results, residual confounding may remain. In particular, the primary PSM model included a limited number of covariates, and additional factors such as COVID-19 severity, socioeconomic status, and detailed immune status may not have been fully captured. Second, our reliance on ICD-10 codes without clinical adjudication (e.g., antibody titers for MG or cerebrospinal fluid analysis for GBS) carries a risk of misclassification. However, prior studies have demonstrated acceptable validity of ICD-10 coding for HZ, with reported positive predictive values exceeding 80% 44, and improved diagnostic accuracy for GBS when supported by objective testing 45. While our NCV utilization data helps mitigate this concern, the potential for overdiagnosis of Bell's palsy in EHR databases must still be acknowledged.

Third, inherent limitations of EHR databases, including potential underdiagnosis and incomplete clinical capture, may affect case ascertainment, as discussed in prior evaluations of the TriNetX platform 46. Fourth, while emerging AI-based diagnostic approaches 38 may provide more granular insights into systemic inflammation, current EHR structures limit our ability to incorporate detailed laboratory or electrophysiological data. Additionally, our vaccine subgroup analysis was limited by low event counts for GBS and MG, and these results should be interpreted with caution. Finally, the geographic distribution of participating healthcare organizations in TriNetX may restrict the generalizability of these findings to uninsured or non-U.S. populations.

## Conclusions

HZ reactivation following COVID-19 is associated with an increased risk of Bell's palsy, GBS, and MG, reflecting distinct temporal patterns suggestive of immediate neurotropic effects and delayed immune-mediated processes. Post-COVID HZ may serve as a clinically relevant marker of neuro-immunological vulnerability, particularly among individuals with metabolic comorbidities. Importantly, COVID-19 vaccination was not associated with an increased risk of these neurological outcomes in our analysis, although subgroup findings should be interpreted with caution, given limited event counts. These findings support a symptom-triggered neurological surveillance approach, with attention to early cranial nerve involvement and delayed neuromuscular manifestations. Further prospective studies are warranted to confirm these associations and clarify the underlying mechanisms.

## Supplementary Material

Supplementary tables.

## Figures and Tables

**Figure 1:**
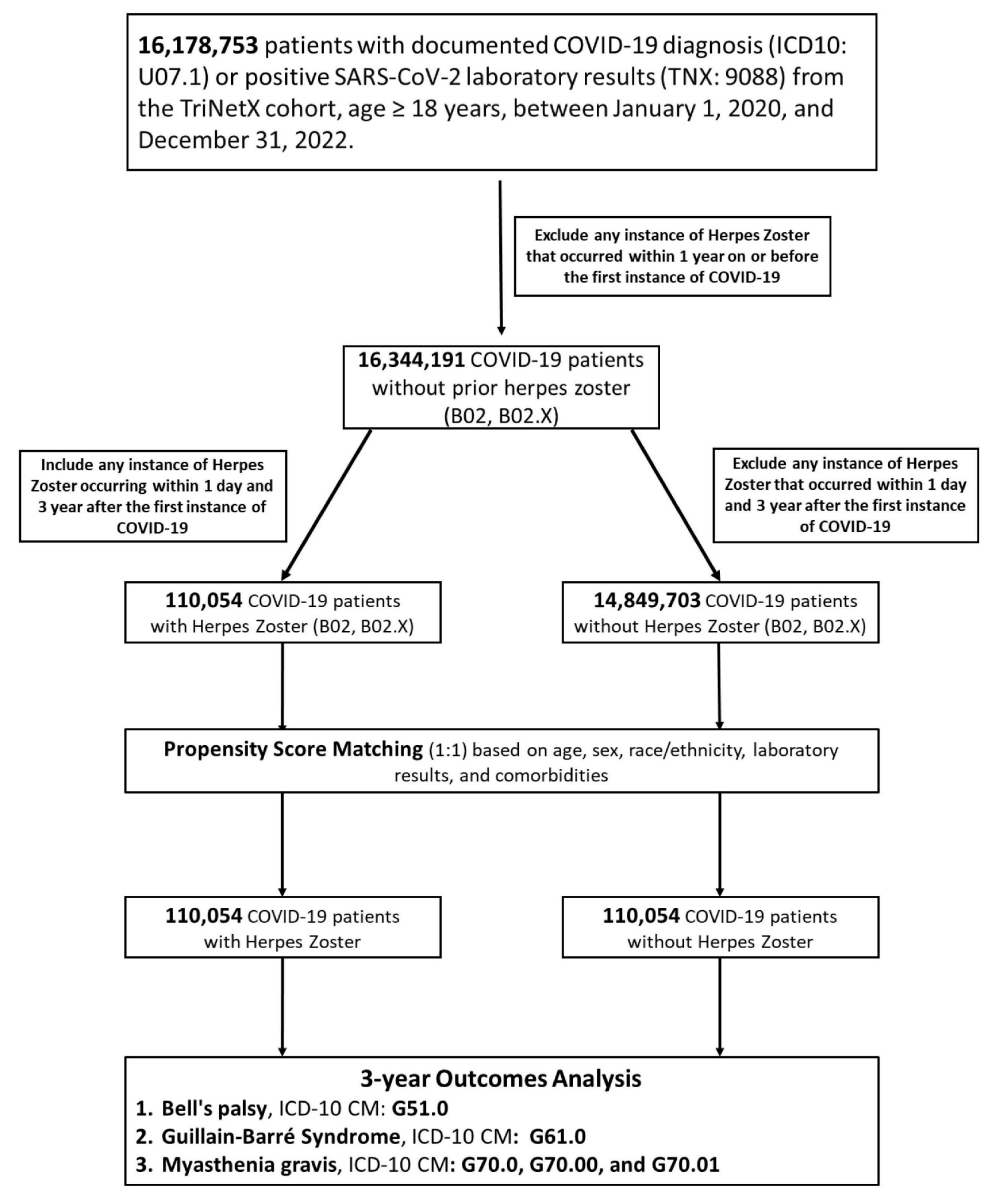
** Flowchart of patient selection and cohort construction.** This diagram illustrates the identification of adult COVID-19 survivors and their subsequent categorization into Herpes Zoster (HZ)-exposed and HZ-unexposed cohorts over a 3-year follow-up period. Exclusion criteria and final cohort sizes (n) after propensity score matching are indicated within the respective steps.

**Table 1:** Baseline characteristics of COVID-19 patients aged 50-59 years with vs without subsequent herpes zoster reactivation, before and after propensity score matching

Characteristic	Before Matching				After Matching			
	Mean±SD	Patient Count	% of Cohort	Std. Dif.	Mean±SD	Patient Count	% of Cohort	Std. Dif.
Demographics								
Age at Index	54.56±2.98 vs 54.45±2.94	16,419 vs 2,251,793	100.00% vs 100.00%	0.0365	54.56±2.98 vs 54.57±2.97	16,418 vs 16,418	100.00% vs 100.00%	0.0043
Female		10,577 vs 1,195,574	64.42% vs 53.09%	0.2316		10,576 vs 10,518	64.42% vs 64.06%	0.0074
Male		5,452 vs 1,020,437	33.20% vs 45.32%	0.2499		5,452 vs 5,498	33.21% vs 33.49%	0.0059
White		11,245 vs 1,302,467	68.49% vs 57.84%	0.2221		11,245 vs 11,346	68.49% vs 69.11%	0.0133
Black or African American		1,981 vs 328,104	12.06% vs 14.57%	0.0738		1,981 vs 2,000	12.07% vs 12.18%	0.0035
Unknown Race		1,559 vs 400,073	9.49% vs 17.77%	0.2428		1,559 vs 1,521	9.50% vs 9.26%	0.0079
Asian		775 vs 90,773	4.72% vs 4.03%	0.0337		774 vs 720	4.71% vs 4.38%	0.0158
Diagnoses								
Hypertensive diseases		4,463 vs 360,007	27.18% vs 15.99%	0.2747		4,462 vs 4,542	27.18% vs 27.66%	0.0109
Diabetes mellitus		2,356 vs 179,437	14.35% vs 7.97%	0.2037		2,355 vs 2,364	14.34% vs 14.40%	0.0016
Ischemic heart diseases		819 vs 70,241	4.99% vs 3.12%	0.0949		819 vs 779	4.99% vs 4.75%	0.0113
Cerebrovascular diseases		301 vs 30,403	1.83% vs 1.35%	0.0386		301 vs 321	1.83% vs 1.96%	0.0089
Laboratory data								
Creatinine	1.04±1.52 vs 1.06±2.39	7,153 vs 610,113	43.56% vs 27.09%	0.0106	1.04±1.52 vs 1.03±2.03	7,152 vs 7,230	43.56% vs 44.04%	0.0055
Glucose	118.49±56.72 vs 117.88±54.70	7,008 vs 606,536	42.68% vs 26.94%	0.0109	118.49±56.72 vs 118.94±56.56	7,008 vs 7,098	42.69% vs 43.23%	0.0079
Calcium	9.36±0.56 vs 9.34±0.57	6,905 vs 575,087	42.05% vs 25.54%	0.0395	9.36±0.56 vs 9.36±0.56	6,904 vs 6,963	42.05% vs 42.41%	0.0032
Urea nitrogen	16.72±10.11 vs 16.37±9.56	6,776 vs 554,166	41.27% vs 24.61%	0.0362	16.72±10.11 vs 16.26±9.57	6,775 vs 6,839	41.27% vs 41.66%	0.0468
Hematocrit	40.08±6.84 vs 39.95±8.14	6,449 vs 556,603	39.28% vs 24.72%	0.0173	40.08±6.84 vs 40.13±7.03	6,448 vs 6,492	39.27% vs 39.54%	0.0083
Hemoglobin	13.35±1.93 vs 13.51±1.99	6,427 vs 552,799	39.14% vs 24.55%	0.0814	13.35±1.93 vs 13.41±1.95	6,426 vs 6,460	39.14% vs 39.35%	0.0309
Alanine aminotransferase	30.57±181.75 vs 30.55±59.50	6,164 vs 506,798	37.54% vs 22.51%	0.0002	30.58±181.77 vs 31.00±101.08	6,163 vs 6,147	37.54% vs 37.44%	0.0029
Aspartate aminotransferase	27.21±44.32 vs 30.09±86.37	6,054 vs 492,589	36.87% vs 21.88%	0.0421	27.21±44.33 vs 30.27±79.43	6,053 vs 6,052	36.87% vs 36.86%	0.0476
Albumin	4.14±0.47 vs 4.12±0.53	5,942 vs 481,307	36.19% vs 21.37%	0.0358	4.14±0.47 vs 4.13±0.51	5,941 vs 5,984	36.19% vs 36.45%	0.0189
Alkaline phosphatase	89.05±47.77 vs 89.57±62.66	5,862 vs 477,000	35.70% vs 21.18%	0.0093	89.05±47.77 vs 91.05±62.52	5,862 vs 5,881	35.70% vs 35.82%	0.0360
Bilirubin, total	0.56±0.88 vs 0.63±1.08	5,768 vs 468,463	35.13% vs 20.80%	0.0685	0.56±0.88 vs 0.60±0.78	5,767 vs 5,799	35.13% vs 35.32%	0.0407
Leukocytes	53.61±432.80 vs 26.08±278.58	5,718 vs 488,168	34.83% vs 21.68%	0.0756	52.84±428.90 vs 25.09±267.03	5,717 vs 5,746	34.82% vs 35.00%	0.0777
Protein	7.08±0.67 vs 7.12±0.77	5,526 vs 455,044	33.66% vs 20.21%	0.0593	7.08±0.67 vs 7.12±0.65	5,526 vs 5,571	33.66% vs 33.93%	0.0580
Platelet mean	9.73±1.33 vs 9.77±1.46	4,074 vs 370,944	24.81% vs 16.47%	0.0292	9.73±1.33 vs 9.77±1.33	4,073 vs 4,051	24.81% vs 24.67%	0.0271
Cholesterol	191.68±45.71 vs 189.25±45.49	3,830 vs 286,158	23.33% vs 12.71%	0.0532	191.68±45.71 vs 188.68±45.18	3,829 vs 3,838	23.32% vs 23.38%	0.0662
Cholesterol in HDL	50.39±20.71 vs 49.37±19.92	3,826 vs 291,274	23.30% vs 12.94%	0.0499	50.39±20.71 vs 50.26±20.13	3,826 vs 3,847	23.30% vs 23.43%	0.0060
Cholesterol in LDL	110.41±38.26 vs 109.73±38.03	3,799 vs 287,023	23.14% vs 12.75%	0.0179	110.42±38.26 vs 108.51±37.87	3,798 vs 3,818	23.13% vs 23.25%	0.0500
Triglyceride	147.07±114.44 vs 147.15±132.31	3,779 vs 291,274	23.02% vs 12.94%	0.0006	147.07±114.44 vs 147.55±143.95	3,779 vs 3,824	23.02% vs 23.29%	0.0037
Hemoglobin A1c	6.74±1.92 vs 6.62±1.81	3,087 vs 243,192	18.80% vs 10.80%	0.0667	6.74±1.92 vs 6.70±1.83	3,086 vs 3,015	18.80% vs 18.36%	0.0237
Magnesium	1.93±0.31 vs 1.94±0.31	1,198 vs 96,288	7.30% vs 4.28%	0.0301	1.93±0.31 vs 1.93±0.31	1,198 vs 1,140	7.30% vs 6.94%	0.0070
Phosphate	3.63±1.08 vs 3.60±1.10	968 vs 70,013	5.90% vs 3.11%	0.0291	3.63±1.08 vs 3.63±1.10	967 vs 883	5.89% vs 5.38%	0.0006
C-reactive protein	17.84±42.43 vs 25.35±52.21	940 vs 73,185	5.72% vs 3.25%	0.1578	17.86±42.45 vs 21.74±49.91	939 vs 905	5.72% vs 5.51%	0.0837
0-10 mg/L		703 vs 48,303	4.3% vs 2.1%	0.121		702 vs 654	4.3% vs 4.0%	0.015
10-20 mg/L		158 vs 12,871	1.0% vs 0.6%	0.045		158 vs 155	1.0% vs 0.9%	0.002
20-30 mg/L		66 vs 6,561	0.4% vs 0.3%	0.019		66 vs 61	0.4% vs 0.4%	0.005
30-60 mg/L		87 vs 9,163	0.5% vs 0.4%	0.018		87 vs 81	0.5% vs 0.5%	0.005
Calcidiol	34.66±16.88 vs 33.97±17.20	793 vs 50,493	4.83% vs 2.24%	0.0403	34.66±16.88 vs 35.75±18.52	793 vs 724	4.83% vs 4.41%	0.0616
Urate	5.82±1.93 vs 5.82±2.02	553 vs 36,237	3.37% vs 1.61%	0.0002	5.82±1.93 vs 5.84±1.97	552 vs 495	3.36% vs 3.02%	0.0082
Medications								
Antilipemic agents		2,521 vs 214,098	15.35% vs 9.51%	0.1779		2,521 vs 2,601	15.36% vs 15.84%	0.0134
Antiarrhythmics		1,916 vs 155,165	11.67% vs 6.89%	0.1653		1,916 vs 1,650	11.67% vs 10.05%	0.0521
Diuretics		1,843 vs 153,195	11.22% vs 6.80%	0.1549		1,843 vs 1,788	11.22% vs 10.89%	0.0107
Beta blockers		1,669 vs 144,071	10.16% vs 6.40%	0.1370		1,669 vs 1,642	10.17% vs 10.00%	0.0055
Calcium channel blockers		1,310 vs 112,927	7.98% vs 5.01%	0.1205		1,310 vs 1,320	7.98% vs 8.04%	0.0022
ACE inhibitors		1,230 vs 113,727	7.49% vs 5.05%	0.1008		1,230 vs 1,254	7.49% vs 7.64%	0.0055
Angiotensin II inhibitor		1,069 vs 88,724	6.51% vs 3.94%	0.1157		1,069 vs 1,114	6.51% vs 6.79%	0.0110

Abbreviations: COVID-19, coronavirus disease 2019; ACE, angiotensin-converting enzyme; SD, standard deviation; St. Diff, standardized difference; values < 0.1 typically indicate good balance.

## Data Availability

Due to licensing and privacy restrictions, the de-identified, aggregate-level data used in this study from the TriNetX Global Health Research Network are not publicly available. TriNetX provides access to data sourced from a global network of healthcare organizations. Researchers may request access through the TriNetX website (https://trinetx.com) or by contacting Privacy@TriNetX.com. Data are also available from the corresponding author upon reasonable request.

## Abbreviations

COVID-19: coronavirus disease 2019
PNS: peripheral nervous system
HZ: herpes zoster
VZV: varicella-zoster virus
HR: hazard ratio
GBS: Guillain-Barré syndrome
MG: myasthenia gravis
EHR: electronic health record
ICD-10-CM: International Classification of Diseases, Tenth Revision, Clinical Modification
STROBE: Strengthening the Reporting of Observational Studies in Epidemiology
IRB: Institutional Review Board
PSM: propensity score matching
SMD: standardized mean difference
RR: relative risk
OR: odds ratio
CI: confidence interval
HIV: human immunodeficiency virus
NCV: nerve conduction velocity
AI: artificial intelligence
