# Supplementary material for: Association Between Post-COVID-19 Herpes Zoster Reactivation and Peripheral Nervous System Disorders: Multinational Real-World Evidence from TriNetX
Source: Int J Med Sci. 2026 Apr 23;23(6):2006–15. doi: 10.7150/ijms.127617 (PMC13181386; doi:10.7150/ijms.127617)
Supplement: Supplementary file 1 — Supplementary tables. [file ijmsv23p2006s1.pdf]

Supplementary Table S1. Three-year Bell's palsy risk: age-restricted cohort (50–59) vs overall cohort (18–89).

This table compares the three-year risk of Bell's palsy between COVID-19 survivors with and without herpes zoster (HZ) reactivation in both the age-restricted cohort (50–59 years) and the overall cohort (18–89 years). Results are presented as unadjusted hazard ratios (HRs) with 95% confidence intervals (CIs) derived from the pre-matching cohort.

Age strata	Outcomes	Cohorts	Patients in cohort	Patients with outcome	Odds Ratio	Survival probability at end of time window	Hazard Ratio	95% CI	Log-Rank test *P*-value
50~59 Years	Bell's palsy	COVID + HZ	21,162	235	4.959	98.817%	4.159	(3.049,5.673)	<0.0001
		COVID - HZ	21,243	48		99.706%			
18~89 years (Entire cohort)		COVID + HZ	109,010	1,095	4.638	98.91%	3.625	(3.151, 4.170)	0.000
		COVID - HZ	109,487	239		99.70%			

Abbreviations: COVID-19, coronavirus disease 2019; HZ, herpes zoster; COVID+HZ, COVID-19 survivors with subsequent herpes zoster reactivation; COVID–HZ, COVID-19 survivors without herpes zoster reactivation; HR, hazard ratio; CI, confidence interval.

Supplementary Table S2. Three-year risks and associations of peripheral nervous system outcomes in COVID-19 survivors with and without herpes zoster reactivation.

This table presents the cumulative incidence, absolute risk differences, and relative measures for Bell's palsy, Guillain–Barré syndrome, and myasthenia gravis among COVID-19 survivors with and without herpes zoster (HZ) reactivation. Results are reported as risk ratios (RRs), odds ratios (ORs), and hazard ratios (HRs) with 95% confidence intervals (CIs) over a three-year follow-up period.

Outcomes	Cohorts	Patients in cohort	Patients with outcome	Risk	Risk Difference	Risk Ratio	Odds Ratio	95% CI	P value
Bell's palsy	COVID + HZ	109,010	1,095	0.010	0.008	4.602	4.638	4.031, 5.336	0.000
	COVID - HZ	109,487	239	0.002					
Guillain–Barré syndrome	COVID + HZ	109,918	79	0.001	0.000	2.325	2.326	1.556, 3.477	0.000
	COVID - HZ	109,975	34	0.000					
Myasthenia gravis	COVID + HZ	109,726	108	0.001	0.001	2.080	2.081	1.494, 2.897	0.000
	COVID - HZ	109,871	52	0.000					

Abbreviations: COVID-19, coronavirus disease 2019; HZ, herpes zoster; COVID+HZ, COVID-19 survivors with subsequent herpes zoster reactivation; COVID–HZ, COVID-19 survivors without herpes zoster reactivation; HR, hazard ratio; CI, confidence interval.

Supplementary Table S3. Landmark analysis of peripheral nervous system outcomes.

This table compares the 1-, 2-, and 3-year risks of Bell's palsy, Guillain–Barré syndrome, and myasthenia gravis between COVID-19 survivors with and without herpes zoster (HZ) reactivation. Results are reported as odds ratios (ORs) and hazard ratios (HRs) with 95% confidence intervals (CIs) at each time point, illustrating temporal patterns of risk over the follow-up period.

Follow up, years	Outcomes	Cohorts	Patients in cohort	Patients with outcome	Odds Ratio	Survival probability at end of time window	Hazard Ratio	95% CI	Log-Rank test *P* value
1	Bell's palsy	COVID + HZ	96,794	422	4.295	99.56%	3.604	(2.896, 4.486)	0.000
		COVID - HZ	97,202	99		99.88%			
2		COVID + HZ	109,010	814	4.754	99.22%	3.828	(3.249, 4.511)	0.000
		COVID - HZ	109,487	173		99.80%			
3		COVID + HZ	109,010	1,095	4.638	98.91%	3.625	(3.151, 4.170)	0.000
		COVID - HZ	109,487	239		99.70%			
1	Guillain-Barré syndrome	COVID + HZ	97,604	33	1.436	99.97%	1.223	(0.718, 2.084)	0.458
		COVID - HZ	97,643	23		99.97%			
2		COVID + HZ	109,918	60	2.502	99.94%	2.066	(1.287, 3.318)	0.002
		COVID - HZ	109,975	24		99.97%			
3		COVID + HZ	109,918	79	2.326	99.92%	1.858	(1.243, 2.779)	0.002
		COVID - HZ	109,975	34		99.96%			
1	Myasthenia gravis	COVID + HZ	97,424	40	1.821	99.96%	1.524	(0.906, 2.564)	0.110
		COVID - HZ	97,566	22		99.97%			
2		COVID + HZ	109,726	80	2.109	99.92%	1.715	(2.139, 4.666)	0.006
		COVID - HZ	109,871	38		99.96%			
3		COVID + HZ	109,726	108	2.081	99.89%	1.640	(1.178, 2.284)	0.003
		COVID - HZ	109,871	52		99.94%			

Abbreviations: COVID-19, coronavirus disease 2019; HZ, herpes zoster; COVID+HZ, COVID-19 survivors with subsequent herpes zoster reactivation; COVID–HZ, COVID-19 survivors without herpes zoster reactivation; HR, hazard ratio; CI, confidence interval.

Supplementary Table S4. Sensitivity analysis of peripheral nervous system outcomes excluding patients with prior immunosuppression.

This table compares the three-year risk of peripheral nervous system outcomes between COVID-19 survivors with and without herpes zoster (HZ) reactivation. Results are presented for the overall cohort and for a sensitivity cohort excluding individuals with documented immunosuppression in the year prior to COVID-19 diagnosis. Data are reported as hazard ratios (HRs) with 95% confidence intervals (CIs).

Exclude immuno-suppression*	Outcomes	Cohorts	Patients in cohort	Patients with outcome	Odds Ratio	Survival probability at end of time window	Hazard Ratio	95% CI	Log-Rank test *P*-value
Yes	Bell's palsy	COVID + HZ	91,892	916	4.879	98.92%	3.744	(3.202, 4.378)	0.000
		COVID - HZ	92,265	190		99.71%			
No (Entire cohort)		COVID + HZ	109,010	1,095	4.638	98.91%	3.625	(3.151, 4.170)	0.000
		COVID - HZ	109,487	239		99.70%			
Yes	Guillain-Barré syndrome	COVID + HZ	92,603	60	3.160	99.93%	2.505	(1.495, 4.198)	0.000
		COVID - HZ	92,625	19		99.97%			
No (Entire cohort)		COVID + HZ	109,918	79	2.326	99.92%	1.858	(1.243, 2.779)	0.002
		COVID - HZ	109,975	34		99.96%			
Yes	Myasthenia gravis	COVID + HZ	92,478	81	1.979	99.91%	1.523	(1.046, 2.217)	0.027
		COVID - HZ	92,591	41		99.94%			
No (Entire cohort)		COVID + HZ	109,726	108	2.081	99.89%	1.640	(1.178, 2.284)	0.003
		COVID - HZ	109,871	52		99.94%			

Abbreviations: COVID-19, coronavirus disease 2019; HZ, herpes zoster; COVID+HZ, COVID-19 survivors with subsequent herpes zoster reactivation; COVID–HZ, COVID-19 survivors without herpes zoster reactivation; HR, hazard ratio; CI, confidence interval.

Supplementary Table S5A. Within-cohort effects of COVID-19 vaccination on peripheral nervous system outcomes.

This table compares the three-year risk of Bell's palsy, Guillain–Barré syndrome, and myasthenia gravis between vaccinated and unvaccinated individuals within COVID-19 survivor cohorts stratified by herpes zoster (HZ) status. Results are reported as hazard ratios (HRs) with 95% confidence intervals (CIs).

Outcomes	Cohort characteristics				KM - survival analysis			
	Cohort (comparison)	Patients in cohort	Patients with outcome	Odds ratio	Survival probability	Hazard ratio	95% CI	Log-Rank test P value
Bell's palsy	COVID-19 + HZ Vaccine (- vs.+)	18,500 vs 18,471	161 vs 183	0.877	99.06% vs 99.98%	0.923	(0.746, 1.140)	0.456
	COVID-19 - HZ Vaccine (- vs.+)	1,115,714 vs 1,113,882	2,080 vs 2,887	0.719	99.73% vs 99.71%	0.922	(0.871, 0.975)	0.005
Guillain-Barré syndrome	COVID-19 + HZ Vaccine (- vs.+)	18,662 vs 18,671	10 vs 15	0.667	99.94% vs 99.92%	0.695	(0.312, 1.548)	0.371
	COVID-19 – HZ Vaccine (- vs.+)	1,120,141 vs 1,120,081	355 vs 390	0.910	99.96% vs 99.96%	1.144	(0.991, 1.321)	0.066
Myasthenia gravis	COVID-19 + HZ Vaccine (- vs.+)	18,642 vs 18,613	17 vs 19	0.893	99.90% vs 99.89%	0.953	(0.495, 1.834)	0.885
	COVID-19 - HZ Vaccine (- vs.+)	1,119,571 vs 1,119,268	496 vs 616	0.805	99.94% vs 99.94%	1.030	(0.915, 1.160)	0.622

Abbreviations: COVID-19, coronavirus disease 2019; HZ, herpes zoster; COVID+HZ, COVID-19 survivors with subsequent herpes zoster reactivation; COVID–HZ, COVID-19 survivors without herpes zoster reactivation; HR, hazard ratio; CI, confidence interval.

Supplementary Table S5B. Association between herpes zoster and peripheral nervous system outcomes stratified by COVID-19 vaccination status.

This table compares the three-year risk of Bell's palsy, Guillain–Barré syndrome, and myasthenia gravis between COVID-19 survivors with and without herpes zoster (HZ) reactivation, stratified by vaccination status. Results are reported as hazard ratios (HRs) with 95% confidence intervals (CIs).

Outcomes	Cohort characteristics				KM - survival analysis			
	Cohort (comparison)	Patients in cohort	Patients with outcome	Odds ratio	Survival probability	Hazard ratio	95% CI	Log-Rank test P value
Bell's palsy	Vaccination C+H vs. C-H	18,471 vs 18,557	183 vs 47	3.941	98.976% vs 99.721%	3.685	(2.674, 5.077)	<0.0001
	No Vaccination C+H vs. C-H	90,535 vs 90,896	912 vs 171	5.399	98.90% vs 99.73%	4.138	(3.514, 4.873)	0.000
Guillain-Barré syndrome	Vaccination C+H vs. C-H	*Based on the outcome terms, the patient count is too small, so detailed results cannot be displayed.*						
	No Vaccination C+H vs. C-H	91,243 vs 91,283	64 vs 29	2.209	99.92% vs 99.96%	1.736	(1.119, 2.693)	0.013
Myasthenia gravis	Vaccination C+H vs. C-H	18,613 vs 18,657	19 vs 14	1.361	99.893% vs 99.919%	1.282	(0.643, 2.557)	0.4797
	No Vaccination C+H vs. C-H	91,109 vs 91,225	89 vs 49	1.819	99.89% vs 99.92%	1.396	(0.985, 1.979)	0.060

Abbreviations: COVID-19, coronavirus disease 2019; HZ, herpes zoster; COVID+HZ, COVID-19 survivors with subsequent herpes zoster reactivation; COVID–HZ, COVID-19 survivors without herpes zoster reactivation; HR, hazard ratio; CI, confidence interval.

Supplementary Table S6. Sensitivity analysis excluding individuals with influenza and HIV infection.

This table compares the three-year risk of peripheral nervous system outcomes between COVID-19 survivors with and without herpes zoster (HZ) reactivation after excluding individuals with influenza or HIV infection within the defined time window. Results are reported as hazard ratios (HRs) with 95% confidence intervals (CIs).

Exclude HIV & Influenza	Outcomes	Cohorts	Patients in cohort	Patients with outcome	Survival probability at end of time window	Hazard Ratio	95% CI	Log-Rank test *P*-value
Yes	Bell's palsy	COVID + HZ	9,390	814	98.914%	4.173	(3.515,4.952)	<0.0001
		COVID - HZ	79,711	156	99.738%			
No (Entire cohort)		COVID + HZ	94,613	996	98.878%	3.607	(3.118,4.172)	<0.0001
		COVID - HZ	95,030	222	99.686%			
Yes	Guillain-Barré syndrome	COVID + HZ	80,024	55	99.928%	2.318	(1.375,3.906)	0.001
		COVID - HZ	80,058	19	99.969%			
No (Entire cohort)		COVID + HZ	95,393	74	99.918%	1.617	(1.089,2.4)	0.016
		COVID - HZ	95,437	37	99.950%			
Yes	Myasthenia gravis	COVID + HZ	79,904	75	99.901%	1.533	(1.041,2.258)	0.029
		COVID - HZ	80,030	39	99.935%			
No (Entire cohort)		COVID + HZ	95,234	98	99.891%	1.542	(1.099,2.164)	0.012
		COVID - HZ	95,392	51	99.929%			

Abbreviations: HZ, herpes zoster; COVID-19, coronavirus disease 2019; COVID+ HZ, COVID-19 survivors with HZ reactivation; COVID- HZ, COVID-19 survivors without HZ reactivation; HR, hazard ratio; CI, confidence interval.

Supplementary Table S7. Comparison of Incident Cases and Diagnostic Frequency for Peripheral Nervous System Disorders in COVID-19 Survivors With and Without Herpes Zoster

This table compares incident cases and total diagnostic counts of peripheral nervous system outcomes between COVID-19 survivors with and without herpes zoster (HZ) reactivation. Results are presented to evaluate potential differences in diagnostic intensity between cohorts.

Outcomes	Characteristic	Cohort	Patients in cohort	Patients with outcome	Average (B/A)	Outcome P value (C+H vs. C-H)
Bell's palsy	(A) Incident case	COVID + HZ	95,784	1,009	1303/1009 = 1.291	p < 0.0001
		COVID - HZ	96,202	190	289/190 = 1.521	
	(B) Number of diagnoses	COVID + HZ	96,695	1,303		p < 0.0001
		COVID - HZ	96,695	289		
Guillain-Barré syndrome	(A) Incident case	COVID + HZ	96,570	76	136/76 = 1.789	p < 0.0001
		COVID - HZ	96,617	30	64/30 = 2.133	
	(B) Number of diagnoses	COVID + HZ	96,695	136		0.0015
		COVID - HZ	96,695	64		
Myasthenia gravis	(A) Incident case	COVID + HZ	96,410	101	323/104 = 3.106	p < 0.0001
		COVID - HZ	96,564	45	130/45 = 2.889	
	(B) Number of diagnoses	COVID + HZ	96,695	323		p < 0.0001
		COVID - HZ	96,695	130		

Abbreviations: HZ, herpes zoster; COVID-19, coronavirus disease 2019; COVID+ HZ, COVID-19 survivors with subsequent HZ reactivation; COVID- HZ, COVID-19 survivors without HZ reactivation.

Supplementary Table S8. Comparison of nerve conduction velocity (NCV) examination utilization.

This table compares the utilization of nerve conduction velocity (NCV) examinations between COVID-19 survivors with and without herpes zoster (HZ) reactivation. Results are presented to evaluate whether increased diagnostic activity may reflect greater clinical evaluation rather than coding-related overdiagnosis.

Outcome	Cohort	Patients in cohort	Patients with outcome	Mean	Standard Deviation	P value
Number of NCV examination	COVID + HZ	96,695	3,845	0.054	0.386	$p < 0.0001$
	COVID - HZ	96,695	1,801	0.022	0.186	

Abbreviations: HZ, herpes zoster; COVID-19, coronavirus disease 2019; COVID+ HZ, COVID-19 survivors with subsequent HZ reactivation; COVID- HZ, COVID-19 survivors without HZ reactivation; NCV, nerve conduction velocity.

Supplementary Table S9. Time-window–based sensitivity analysis by timing of herpes zoster onset.

This sensitivity analysis assesses potential immortal time bias by categorizing the timing of HZ onset after the COVID-19 index date into specific windows (6, 12, 24, and 36 months). For each time window, a separate 1:1 propensity score-matched cohort was constructed.

Incident HZ after COVID-19	Outcomes	Cohorts	Patients in cohort	Patients with outcome	Survival probability at end of time window	Hazard Ratio	95% CI	Log-Rank test P value
6 months	Bell's palsy	COVID + HZ	20,978	243	98.765%	5.02	(3.629,6.942)	<0.0001
		COVID - HZ	21,100	43	99.723%			
12 months		COVID + HZ	37,608	408	98.84%	4.332	(3.413, 5.499)	<0.0001
		COVID - HZ	37,803	81	99.70%			
24 months		COVID + HZ	67,563	729	98.85%	4.279	(3.573, 5.125)	<0.0001
		COVID - HZ	67,866	141	99.72%			
36 months		COVID + HZ	94,613	996	98.878%	3.607	(3.118,4.172)	<0.0001
		COVID - HZ	95,030	222	99.686%			
6 months	Guillain-Barré syndrome	COVID + HZ	Based on the outcome terms, the patient count is too small, so detailed results cannot be displayed.					Nil
		COVID - HZ						
12 months		COVID + HZ	37,945	27	99.92%	2.302	(1.114, 4.756)	0.020
		COVID - HZ	37,967	10	99.97%			
24 months		COVID + HZ	68,130	49	99.92%	2.027	(1.205, 3.409)	0.007
		COVID - HZ	68,162	20	99.96%			
36 months		COVID + HZ	95,393	74	99.918%	1.617	(1.089,2.4)	0.016
		COVID - HZ	95,437	37	99.950%			
6 months	Myasthenia gravis	COVID + HZ	21,144	14	99.923%	0.637	(0.32,1.271)	0.197
		COVID - HZ	21,173	19	99.878%			
12 months		COVID + HZ	37,890	33	99.90%	1.069	(0.639, 1.787)	0.800
		COVID - HZ	37,938	26	99.91%			
24 months		COVID + HZ	68,022	66	99.90%	1.311	(0.888, 1.936)	0.172
		COVID - HZ	68,120	41	99.92%			
36 months		COVID + HZ	95,234	98	99.891%	1.542	(1.099,2.164)	0.012
		COVID - HZ	95,392	51	99.929%			

Abbreviations: HZ, herpes zoster; COVID-19, coronavirus disease 2019; COVID+ HZ, COVID-19 survivors with subsequent HZ reactivation; COVID- HZ, COVID-19 survivors without HZ reactivation; HR, hazard ratio; CI, confidence interval.

Supplementary Table S10. Sensitivity Analysis Excluding Individuals With Peripheral Nervous System Outcomes Occurring Prior to or Concurrent With the First Recorded Herpes Zoster Episode.

This table compares the three-year risk of peripheral nervous system outcomes between COVID-19 survivors with and without herpes zoster (HZ) reactivation after excluding individuals whose outcomes occurred prior to or on the same date as the first recorded HZ episode. Results are presented alongside the entire cohort to assess the temporal relationship between HZ exposure and outcome occurrence. Hazard ratios (HRs) with 95% confidence intervals (CIs) are reported.

Exclude PNS before first HZ	Outcomes	Cohorts	Patients in cohort	Patients with outcome	Survival probability at end of time window	Hazard Ratio	95% CI	Log-Rank test *P*-value
Yes	Bell's palsy	COVID + HZ	94,560	367	99.579%	1.428	(1.203,1.696)	<0.0001
		COVID - HZ	94,494	202	99.713%			
No (Entire cohort)		COVID + HZ	94,613	996	98.878%	3.607	(3.118,4.172)	<0.0001
		COVID - HZ	95,030	222	99.686%			
Yes	Guillain-Barré syndrome	COVID + HZ	94,948	30	99.966%	0.701	(0.429,1.146)	0.155
		COVID - HZ	94,924	34	99.953%			
No (Entire cohort)		COVID + HZ	95,393	74	99.918%	1.617	(1.089,2.4)	0.016
		COVID - HZ	95,437	37	99.950%			
Yes	Myasthenia gravis	COVID + HZ	94,946	35	99.96%	0.460	(0.303,0.698)	0.0002
		COVID - HZ	94,863	60	99.916%			
No (Entire cohort)		COVID + HZ	95,234	98	99.891%	1.542	(1.099,2.164)	0.012
		COVID - HZ	95,392	51	99.929%			

Abbreviations: COVID-19, coronavirus disease 2019; HZ, herpes zoster; PNS, peripheral nervous system; HR, hazard ratio; CI, confidence interval.

Supplementary Table S11. Comparison of Hospitalization Burden between COVID-19 Survivors with and Without Herpes Zoster

This table compares the number of hospitalizations between COVID-19 survivors with and without HZ reactivation. Results are presented as mean and standard deviation (SD) to assess differences in healthcare utilization between cohorts.

Outcome	Cohort	Patients in cohort	Patients with outcome	Mean	Standard Deviation	P value
Number of Hospitalization	COVID + HZ	96,695	40,295	2.532	6.98	p < 0.0001
	COVID - HZ	96,695	30,546	1.446	4.717	

Abbreviations: HZ, herpes zoster; COVID-19, coronavirus disease 2019; COVID+ HZ, COVID-19 survivors with subsequent HZ reactivation; COVID- HZ, COVID-19 survivors without HZ reactivation.

Supplementary Table S12. Impact of Anti-Herpes Zoster Therapy on 3-Year Incidence of Bell's palsy in Patients with Concurrent COVID-19 and Herpes Zoster

This table compares the 3-year risk of Bell's palsy between patients with and without anti-herpes zoster therapy among COVID-19 survivors with herpes zoster (HZ). Results are presented using Kaplan–Meier analysis and hazard ratios (HRs) with 95% confidence intervals (CIs).

Outcomes	Cohort characteristics: COVID-19 + HZ				KM - survival analysis			
	Cohort	Patients in cohort	Patients with outcome	Odds ratio	Survival probability	Hazard ratio	95% CI	Log-Rank test P value
Bell's palsy	+ Anti-HZ	1,661	27	1.366	98.203%	1.378	(0.773,2.457)	0.275
	- Anti-HZ	1,673	20		98.729%			

Abbreviations: HZ, herpes zoster; COVID-19, coronavirus disease 2019; + Anti-HZ, received anti-herpes zoster therapy; - Anti-HZ, did not receive anti-herpes zoster therapy; HR, hazard ratio; CI, confidence interval.

Supplementary Table S13. Sensitivity analysis of herpes zoster vaccination and Bell's palsy risk.

This table evaluates the impact of herpes zoster (HZ) vaccination on the 3-year risk of Bell's palsy. Results for Guillain–Barré syndrome and myasthenia gravis were not estimable due to insufficient event counts.

Exclude HZ vaccination	Outcomes	Cohorts	Patients in cohort	Patients with outcome	Survival probability at end of time window	Hazard Ratio	95% CI	Log-Rank test *P*-value
Yes	Bell's palsy	COVID + HZ	2,710	33	98.724%	0.951	(0.593,1.525)	0.8338
No		COVID + HZ	2,707	36	98.653%			

Abbreviations: HZ, herpes zoster; COVID-19, coronavirus disease 2019; COVID+ HZ, COVID-19 survivors with subsequent HZ reactivation; HR, hazard ratio; CI, confidence interval.

Supplementary Table S14. E-value analysis for unmeasured confounding in peripheral nervous system outcomes.

This table presents E-values for the observed associations to assess the robustness of the results to potential unmeasured confounding.

Outcomes	Cohorts	Hazard Ratio	95% CI	Hazard Ratio E-value	CI E-value
Bell's palsy	COVID + HZ	3.607	(3.118,4.172)	6.674	5.688
	COVID - HZ				
Guillain-Barré syndrome	COVID + HZ	1.617	(1.089,2.4)	2.616	1.400
	COVID - HZ				
Myasthenia gravis	COVID + HZ	1.542	(1.099,2.164)	2.456	1.429
	COVID - HZ				

Abbreviations: HZ, herpes zoster; COVID-19, coronavirus disease 2019; COVID+ HZ, COVID-19 survivors with subsequent HZ reactivation; COVID- HZ, COVID-19 survivors without HZ reactivation; CI, confidence interval.
